# Identification of the Best Semantic Expansion to Query PubMed Through Automatic Performance Assessment of Four Search Strategies on All Medical Subject Heading Descriptors: Comparative Study

**DOI:** 10.2196/12799

**Published:** 2020-06-04

**Authors:** Clément R Massonnaud, Gaétan Kerdelhué, Julien Grosjean, Romain Lelong, Nicolas Griffon, Stefan J Darmoni

**Affiliations:** 1 Department of Biomedical Informatics Rouen University Hospital Rouen France; 2 Laboratoire d'Informatique Médicale et d'Ingénierie des Connaissances en e-Santé, U1142, INSERM Sorbonne Université Paris France

**Keywords:** bibliographic database, information retrieval, literature search, Medical Subject Headings, MEDLINE, PubMed, precision, recall, search strategy, thesaurus

## Abstract

**Background:**

With the continuous expansion of available biomedical data, efficient and effective information retrieval has become of utmost importance. Semantic expansion of queries using synonyms may improve information retrieval.

**Objective:**

The aim of this study was to automatically construct and evaluate expanded PubMed queries of the form *“preferred term”[MH] OR “preferred term”[TIAB] OR “synonym 1”[TIAB] OR “synonym 2”[TIAB] OR …,* for each of the 28,313 Medical Subject Heading (MeSH) descriptors, by using different semantic expansion strategies. We sought to propose an innovative method that could automatically evaluate these strategies, based on the three main metrics used in information science (precision, recall, and F-measure).

**Methods:**

Three semantic expansion strategies were assessed. They differed by the synonyms used to build the queries as follows: MeSH synonyms, Unified Medical Language System (UMLS) mappings, and custom mappings (Catalogue et Index des Sites Médicaux de langue Française [CISMeF]). The precision, recall, and F-measure metrics were automatically computed for the three strategies and for the standard automatic term mapping (ATM) of PubMed. The method to automatically compute the metrics involved computing the number of all relevant citations (A), using National Library of Medicine indexing as the gold standard (*“preferred term”[MH]*), the number of citations retrieved by the added terms (*”synonym 1“[TIAB] OR ”synonym 2“[TIAB] OR …*) (B), and the number of relevant citations retrieved by the added terms (combining the previous two queries with an “AND” operator) (C). It was possible to programmatically compute the metrics for each strategy using each of the 28,313 MeSH descriptors as a “preferred term,” corresponding to 239,724 different queries built and sent to the PubMed application program interface. The four search strategies were ranked and compared for each metric.

**Results:**

ATM had the worst performance for all three metrics among the four strategies. The MeSH strategy had the best mean precision (51%, SD 23%). The UMLS strategy had the best recall and F-measure (41%, SD 31% and 36%, SD 24%, respectively). CISMeF had the second best recall and F-measure (40%, SD 31% and 35%, SD 24%, respectively). However, considering a cutoff of 5%, CISMeF had better precision than UMLS for 1180 descriptors, better recall for 793 descriptors, and better F-measure for 678 descriptors.

**Conclusions:**

This study highlights the importance of using semantic expansion strategies to improve information retrieval. However, the performances of a given strategy, relatively to another, varied greatly depending on the MeSH descriptor. These results confirm there is no ideal search strategy for all descriptors. Different semantic expansions should be used depending on the descriptor and the user’s objectives. Thus, we developed an interface that allows users to input a descriptor and then proposes the best semantic expansion to maximize the three main metrics (precision, recall, and F-measure).

## Introduction

### Background

With the continuous expansion of available biomedical data, efficient and effective information retrieval has become extremely important. The number of citations for biomedical literature accessible through the PubMed search engine, a service of the US National Library of Medicine (NLM), was over 30 million in January 2019, and of these, more than 26 million were from the MEDLINE database. The number of citations added to MEDLINE each year now exceeds 1 million (1,178,360 citations added in 2016) [1]. PubMed is one of the most used tools to access these data, and its popularity is growing steadily each year (from 2.5 billion searches performed in 2013 to 3.3 billion in 2017) [2].

However, numerous studies have reported that users lack search skills for the effective use of PubMed [3-5]. Although a basic search using PubMed can be relatively straightforward, a deeper understanding of its structure and underlying search algorithm is needed to perform an effective search of the literature. In order to improve the accuracy of information retrieval, MEDLINE citations are indexed in the Medical Subject Headings (MeSH) thesaurus [6], but most users do not know it well enough and do not commonly use its descriptors to build their queries [7,8]. The MeSH thesaurus, developed by the NLM, is a list of descriptors covering the biomedical field. Moreover, users rarely employ search tags and therefore do not fully exploit the features of PubMed [9]. Consequently, the NLM has developed an automatic process to modify users’ explicit queries called automatic term mapping (ATM). Entry terms are mapped to their corresponding MeSH descriptors and compound words are broken down and combined with the Boolean operators “AND” and “OR” and searched with the tag [All fields] [10].

Comprehensive literature searching requires the use of both bibliographic database searching and diverse supplementary search methods [11,12]. The purpose of ATM is to improve information retrieval in bibliographic database searching, but several studies have proposed alternative processes to enhance users’ queries that have yielded better results. For instance, Kim et al proposed the use of semantic techniques, such as document similarity [13]. PubMed has also implemented the recommendation of a “related articles” feature [14]. This feature uses the PubMed-related citations algorithm, which is a probabilistic topic-based model developed by Lin and Wilbur [15]. Wei et al proposed a strategy to enhance this feature [16]. Additionally, Afzal et al proposed methods for the automation of query generation [17,18]. Other popular strategies propose to perform semantic expansion with synonyms of the entry terms. These strategies vary in the knowledge organization system (KOS) they use to perform the expansions. Aronson et al in 1997 [19] and Hersh et al in 2000 [20] proposed the use of Unified Medical Language System (UMLS) mappings to perform query expansion. The UMLS thesaurus maps terms of different KOSs using concept unique identifiers (CUIs). In 2009, Thirion et al proposed the expansion of queries with MeSH synonyms [21]. This strategy was also explored in 2016 by Wright et al [22]. In the MeSH thesaurus, each descriptor has a preferred term and may have some synonyms. This optimization led to a great improvement in the performance of information retrieval. In 2012, Griffon et al proposed the use of the UMLS to perform the expansion [23], leading to a slight increase in recall but a decrease in precision. Among other strategies, Xu et al [24] proposed a biomedical query expansion framework based on learning-to-rank methods, in which term-ranking models are trained to refine the candidate expansion terms by selecting the most relevant terms for enriching the original query.

### Prior Work

In order to improve information retrieval, our team (physicians, librarians, and terminology specialists) developed a new strategy of semantic expansion using new mappings between various KOSs, which was called CISMeF (French acronym, Catalogue et Index des Sites Médicaux de langue Française) mappings. Health Terminology/Ontology Portal (HeTOP) is a cross-lingual multi-terminology server also developed by the CISMeF team, which contains 86 KOSs in 45 languages. The CISMeF mappings are mappings between terms of these 86 KOSs. The mappings were created in various ways. Some concepts were mapped automatically, using UMLS CUIs. However, most of the KOSs included in HeTOP are not included in the UMLS (65 out of the 86 KOSs). For these KOSs, the terms were mapped automatically using natural language processing or manually by librarians and KOS specialists. Moreover, some of the automatically mapped terms were verified and manually curated by librarians and KOS specialists (supervised mappings). Information on HeTOP and CISMeF mappings has been detailed in a previous paper [25]. The performance of this new kind of semantic expansion using CISMeF mappings was manually assessed in a previous study by Massonnaud et al [26].

Although these different strategies have greatly improved the effectiveness of information retrieval, there are limitations to the assessment of their performances. Assessments were performed manually, allowing only small samples of descriptors and citations. Moreover, all the studies revealed a great variability in results depending on the descriptor used. This behavior suggests that there is no semantic expansion that would be optimal for all descriptors and that the semantic expansion to be used should be chosen according to the specific descriptor and the user’s objective (ie, when seeking either better precision or recall or a harmonic mean view using F-measure).

### Objective

The aim of this study was to automatically construct and evaluate expanded PubMed queries of the form *“preferred term”[MH] OR “preferred term”[TIAB] OR “synonym 1”[TIAB] OR “synonym 2”[TIAB] OR …*, for each of the 28,313 MeSH descriptors by using different semantic expansion strategies. We sought to propose an innovative method that could automatically evaluate these strategies, based on the three main metrics used in information science (precision, recall, and F-measure).

## Methods

### Semantic Expansion Strategies

Four strategies were assessed in this study, including the standard ATM of PubMed and three kinds of queries enhanced with semantic expansions (MeSH, UMLS, and CISMeF). The three semantic expansion strategies differed in the set of synonyms used to expand the query. For example, for the MeSH descriptor ”diabetes mellitus, type 2“[MH], the query built with the ATM strategy was as follows: *(”diabetes mellitus, type 2“[MeSH Terms] OR ”type 2 diabetes mellitus“[All Fields] OR ”diabetes mellitus, type 2“[All Fields])*, and the query built with the UMLS strategy was as follows: *(”diabetes mellitus, type 2“[MH] OR ”adult onset diabetes“[TIAB] OR ”adult onset diabetes mellitus“[TIAB] OR ”adult-onset diabetes“[TIAB] OR ”adult-onset diabetes mellitus“[TIAB] OR …).* The four strategies were applied for each of the 28,313 MeSH descriptors, and their respective performance was assessed by computing standard metrics (precision, recall, and F-measure).

### Automatic Metrics Computation

**Figure 1:**
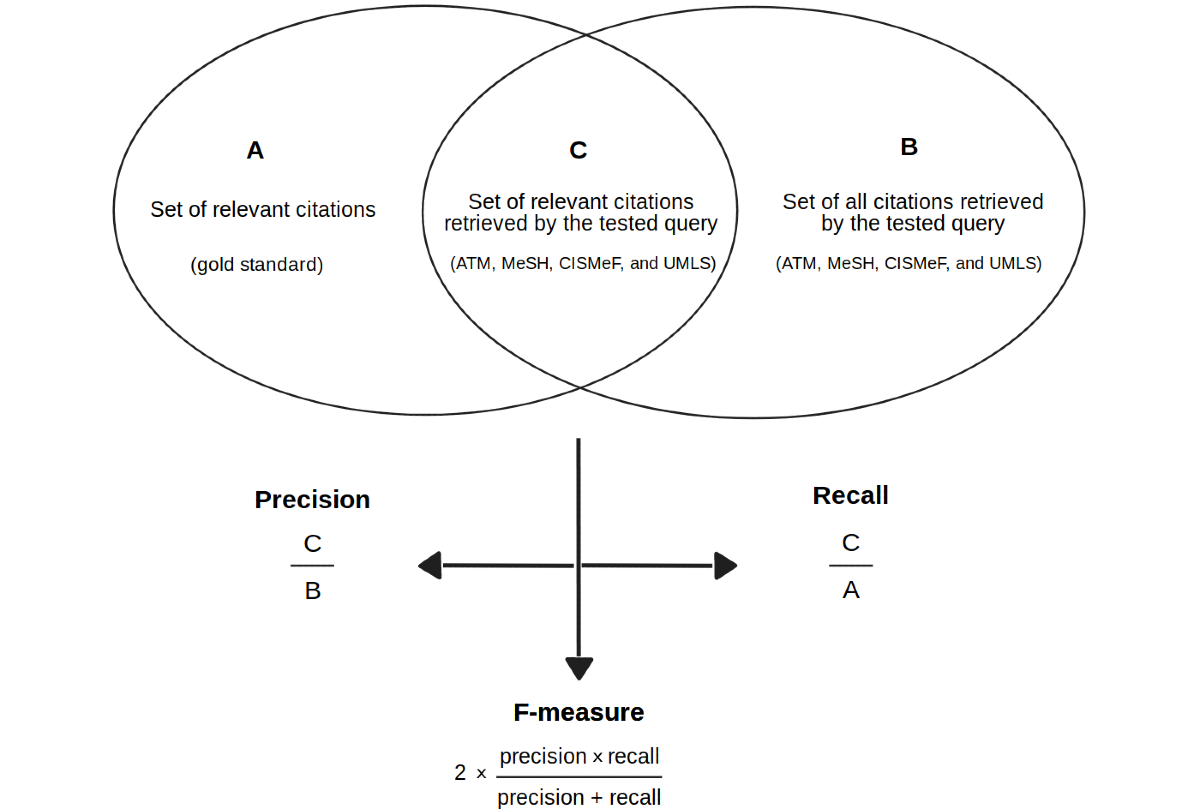
Representation of the three sets of citations retrieved for each descriptor, and how they were used to compute the metrics. ATM: automatic term mapping; CISMeF: Catalogue et Index des Sites Médicaux de langue Française; MeSH: Medical Subject Headings; UMLS: Unified Medical Language System.

Figure 1 depicts how the metrics were computed. Precision was defined as the fraction of relevant citations among the retrieved citations. Recall was defined as the fraction of relevant citations retrieved from the total number of relevant citations. The traditional F-measure (or F1 score) was defined as the harmonic mean of the precision and the recall, and the value is provided by the following formula: 2 × (precision × recall) / (precision + recall). Therefore, in order to automatically estimate these metrics for a given query and a given descriptor, it was necessary to identify the set of all relevant citations for the descriptor (A; ie, the gold standard), the set of all citations retrieved by the query (B), and the set of citations retrieved by the query that were relevant (C; ie, the intersection of A and B).

The set of relevant citations (A) was defined using NLM’s indexing as the gold standard. For a query built from a particular MeSH descriptor, a citation was considered relevant if it was indexed with that same descriptor. Therefore, the total number of relevant citations (A) was retrieved via the following query: *“preferred term”[MH].* The queries for set (B) were constructed as follows: for the ATM strategy, the query was constructed with *“preferred term”[TIAB].* For the other three strategies, the query was constructed with the synonyms retrieved by the expansion strategy (*“synonym 1”[TIAB] OR “synonym 2”[TIAB] OR etc.*). Consequently, the number of relevant citations retrieved (C) could be computed by combining the previous two queries with an “AND” operator as follows: *(“preferred term”[MH] AND “preferred term”[TIAB])* for ATM and *(“preferred term”[MH] AND (*“*synonym 1”[TIAB] OR “synonym 2”[TIAB] OR etc.))* for the three other strategies. The tag *[All fields]* was replaced with *[TIAB]* since *[All fields]* also searches the indexation field of the citations, therefore conflicting with the *[MH]* tag. Moreover, the scope of the search was reduced to indexed citations by adding the tag *medline[sb]* so that all queries were performed on the same set of manually indexed citations*.* Table 1 shows a summary of the syntax of the resulting nine different queries.

**Table 1:** Summary of the syntax of the nine different queries used in this study.

Strategy	Relevant citations (A)	Retrieved citations (B)	Relevant citations retrieved (C)
ATM^a^	”pref. term“[MH]	”pref. term“[TIAB] AND medline[sb]	”pref. term“[MH] AND (”pref. term“[TIAB]) AND medline[sb]
MeSH^b^	”pref. term“[MH]	(”MeSH synonym 1“[TIAB] OR ”MeSH synonym 2“[TIAB] OR …) AND medline[sb]	”pref. term“[MH] AND (”MeSH synonym 1“[TIAB] OR ”MeSH synonym 2“[TIAB] OR …) AND medline[sb]
UMLS^c^	”pref. term“[MH]	(”UMLS synonym 1“[TIAB] OR ”UMLS synonym 2“[TIAB] OR …) AND medline[sb]	”pref. term“[MH] AND (”UMLS synonym 1“[TIAB] OR ”UMLS synonym 2“[TIAB] OR …) AND medline[sb]
CISMeF^d^	”pref. term“[MH]	(”CISMeF synonym 1“[TIAB] OR ”CISMeF synonym 2“[TIAB] OR …) AND medline[sb]	”pref. term“[MH] AND (”CISMeF synonym 1“[TIAB] OR ”CISMeF synonym 2“[TIAB] OR …) AND medline[sb]

^a^ATM: automatic term mapping.

^b^MeSH: Medical Subject Headings.

^c^UMLS: Unified Medical Language System.

^d^CISMeF: Catalogue et Index des Sites Médicaux de langue Française.

### Data Collection

Initially, the queries were built using the HeTOP terminology server [25], which provides relations between multiple KOSs. Given a particular concept, it is possible to automatically gather the MeSH preferred term of this concept and its synonyms from the KOS of interest. As the 2018 version of the MeSH was not released at the time of this study, the 2017 version containing 28,313 descriptors was used, and of these descriptors, 26,636 were used at least once for indexing citations. It was then possible to programmatically build the nine different types of queries (Table 1) for each of the 26,636 descriptors, resulting in a total of 239,724 queries. ATM’s behavior regarding the split of compound words was reproduced exactly. In order to shorten the length of the queries, the terms were set to lowercase and multiple occurrences of the exact same term were removed. Thereafter, the citation count for each of the 239,724 queries was retrieved via PubMed’s application programming interface. The processing time of these 239,724 queries on a microcomputer was around 3 hours and 30 minutes. Therefore, it is scalable and can be run frequently.

### Statistical Analysis

The mean precision, recall, and F-measure were computed for the 26,636 descriptors and for each of the four search strategies. The four strategies were ranked, and the number of descriptors for which the CISMeF strategy had better results than each of the three other strategies was computed, considering a difference of at least 5% (arbitrary). The metrics were also computed with stratification according to the MeSH category. Statistical analysis was performed using R software (version 3.4.3; R Foundation for Statistical Computing, Vienna, Austria). As the analysis was performed on the entire set of MeSH descriptors, confidence intervals and *P* values were not needed and therefore not computed.

## Results

Table 2 shows the mean precision, recall, and F-measure for each of the four search strategies. ATM had the worst performance for all three metrics among the four strategies. MeSH had the best mean precision (51%, SD 23%). CISMeF and UMLS had identical results for precision. UMLS had the best recall and F-measure (41% and 36%, respectively). CISMeF had the second best recall and F-measure.

Table 3 shows the number of descriptors for which two strategies had equal precision, recall, or F-measure. Table 4 shows the number of descriptors for which the metric score of a strategy was at least 5% better than another strategy.

**Table 2:** Mean performances of the four search strategies for the 26,636 Medical Subject Heading descriptors.

KOS^a^	Precision (%), mean (SD)	Recall (%), mean (SD)	F-measure (%), mean (SD)
ATM^b^	44 (24)	31 (29)	28 (23)
MeSH^c^	51 (23)	38 (31)	35 (24)
CISMeF^d^	49 (23)	40 (31)	35 (24)
UMLS^e^	49 (23)	46 (31)	36 (24)

^a^KOS: knowledge organization system.

^b^ATM: automatic term mapping.

^c^MeSH: Medical Subject Headings.

^d^CISMeF: Catalogue et Index des Sites Médicaux de langue Française.

^e^UMLS: Unified Medical Language System.

**Table 3:** Number of descriptors for which two strategies had equal precision, recall, or F-measure.

KOS^a^	Precision, n	Recall, n	F-measure, n
ATM^b^ and MeSH^c^	3037	3959	2938
ATM and CISMeF^d^	2551	3410	2459
ATM and UMLS^e^	2409	3265	2320
MeSH and CISMeF	19,261	20,232	19,001
MeSH and UMLS	17,176	18,394	16,917
CISMeF and UMLS	18,819	19,956	18,565

^a^KOS: knowledge organization system.

^b^ATM: automatic term mapping.

^c^MeSH: Medical Subject Headings.

^d^CISMeF: Catalogue et Index des Sites Médicaux de langue Française.

^e^UMLS: Unified Medical Language System.

**Table 4:** Comparisons of the strategies for each metric.

Strategy comparison	Metric, n^a^		
	Precision	Recall	F-measure
CISMeF^b^ vs UMLS^c^	1180 vs 1017	793 vs 1262	678 vs 1140
MeSH^d^ vs UMLS	2285 vs 215	9 vs 2857	553 vs 1761
CISMeF vs MeSH	170 vs 2088	2372 vs 9	1403 vs 669
MeSH vs ATM^e^	9650 vs 3299	8198 vs 3404	8150 vs 2446
CISMeF vs ATM	9112 vs 4557	9724 vs 2949	8895 vs 2628
ATM vs UMLS	4682 vs 9094	2852 vs 10,047	2448 vs 9217

^a^The numbers are the numbers of descriptors for which the metric score of a strategy was at least 5% better than another strategy.

^b^CISMeF: Catalogue et Index des Sites Médicaux de langue Française.

^c^UMLS: Unified Medical Language System.

^d^MeSH: Medical Subject Headings.

^e^ATM: automatic term mapping.

**Figure 2:**
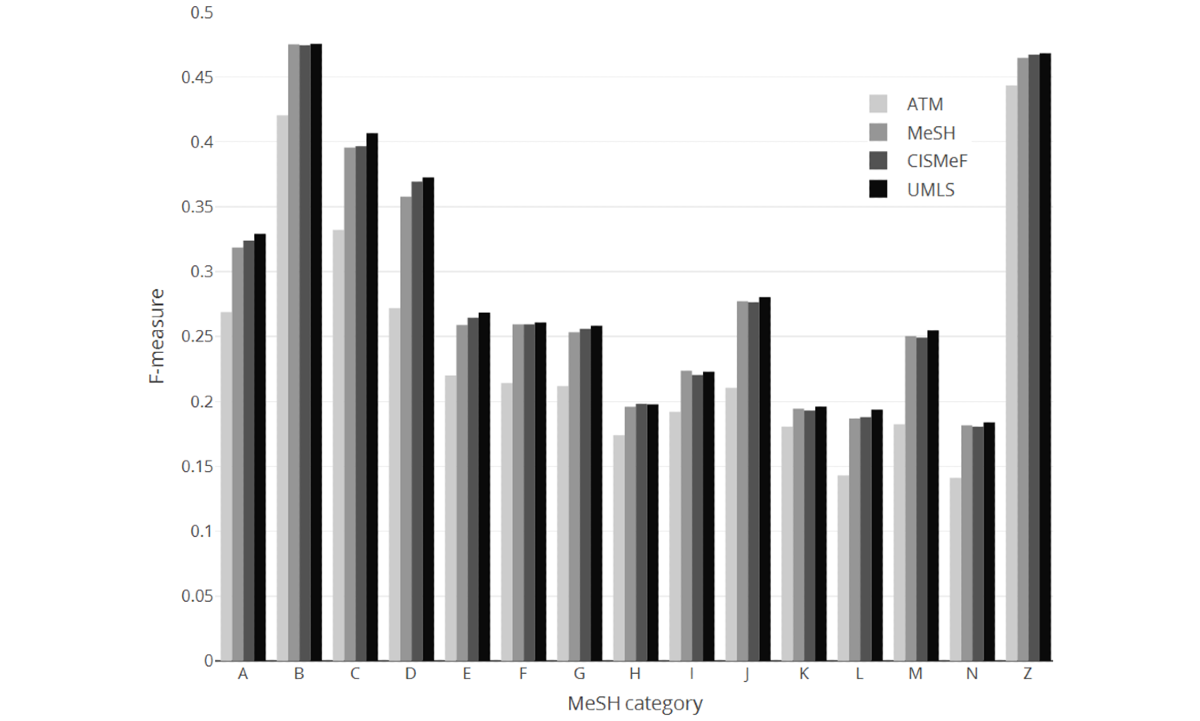
F-measure scores of the four search strategies depending on the MeSH category of the descriptor. ATM: automatic term mapping; CISMeF: Catalogue et Index des Sites Médicaux de langue Française; MeSH: Medical Subject Headings; UMLS: Unified Medical Language System.

The analysis stratified according to the category (tree) of the MeSH descriptor revealed the same trends for all three metrics. The best precision was obtained with category C (diseases) by the MeSH strategy (58.16%). MeSH had the best precision for all categories expect category B (organisms), for which ATM had the best precision (data not shown). The best recall was obtained with category B by UMLS (64.28%), which had the best results for 11 out of the 15 categories. CISMeF had the best recall for the following remaining four categories: H (disciplines and occupations), K (humanities), L (information science), and N (health care). ATM had the worst recall for all categories (data not shown). Figure 2 shows the F-measure scores for each of the four strategies depending on the MeSH category of the descriptor. The best F-measure was obtained with category B by UMLS (47.55%). UMLS had the best F-measure for all categories except category H, for which CISMeF had the best score (19.82%), and category I (anthropology, education, sociology, and social phenomena), for which MeSH had the best F-measure (22.38%).

## Discussion

### Principal Findings

Of the four strategies assessed in this study, PubMed’s standard ATM had the worst mean performances for the three metrics measured (ie, precision, recall, and F-measure). These results are consistent with the findings of previous studies [21,23,26]. The best precision was obtained with the MeSH strategy (50.93%). The mean values of precision for both the CISMeF and UMLS strategies were identical (49.20%). For recall and F-measure, the best performance was obtained by the UMLS strategy, followed by the CISMeF strategy and MeSH strategy.

Even though the differences between the mean performances of the three enhanced strategies (ie, MeSH, CISMeF, and UMLS) were small, with no difference at all for numerous descriptors, the finding did not reflect the important variability of the results. Indeed, for each metric, the ranking of the four strategies was greatly dependent on the descriptor. For instance, although UMLS and CISMeF had identical performances for mean precision, CISMeF had better precision than UMLS for 1180 descriptors. Likewise, the CISMeF strategy had better recall for 793 descriptors and better F-measure for 678 descriptors. Even the ATM strategy, which had much lower mean results for all three metrics, was ranked first for several descriptors (data not shown).

The important variability of the performances found here is consistent with the results of previous studies [21,23,26]. This variability was an important limiting factor for these studies since the assessments were performed manually and therefore on a restricted set of descriptors. Consequently, the interpretation of the results was difficult and had important limitations. The objective of this study was to implement and evaluate an original approach for the automatic assessment of the three main metrics of information science. This innovative method allowed us to test the four different strategies of semantic expansion on the entire set of MeSH preferred descriptors (n=28,313) rather than on a small subset. The results found with this new method conform to those of previous studies, with a similar ranking of the four search strategies. Working out the reasons for variability would be a complex endeavor, as they differ from one descriptor to another. The first obvious reason for a loss of precision, for instance, is an increased number of synonyms used. A higher number of terms in a query is associated with a higher likelihood of it having less precision. For example, the CISMeF expansion for the term “kidney tubular necrosis, acute” had a precision of 97.4% as against 36.6% for the UMLS expansion. The CISMeF expansion included eight terms, whereas the UMLS expansion included 18 terms. Even with a comparable number of terms, a loss in precision could be caused by a single term in the query, for instance, an acronym or a broader synonym (hyperonym). For example, the CISMeF expansion for the term “drug interactions” had a precision of 56.3% as against 5.1% for the UMLS expansion. Both expansions are based on slight variations of the combination of “drug” and “interactions,” but one term in the UMLS expansion is simply “interactions,” which leads to a lot of noise and thus a decrease in the precision of the query. For recall, the reasons for variation are somewhat reciprocal. For instance, the CISMeF expansion for the term “chronic disease” yields a recall of 73.8% as against 7.8% for the UMLS expansion. Both expansions use variations of the combination of “chronic” and “disease,” but the CISMeF expansion uses an additional “chronic” term alone. However, this gain in recall is at the cost of a loss in precision (13.1% loss).

The ranking of the four strategies was similar after stratification according to the descriptor’s category in the MeSH tree. The exact same evaluation was performed with different tags in the queries [14]. The assessment was also performed over different time intervals. The tag *[majr] was tested instead of *[mh], and all strategies were tested with and without the explosion behavior. The explosion is activated by default in PubMed’s ATM, but it was not feasible to reproduce the explosion in our expanded queries, as this would have resulted in too large queries. This could slightly bias the results for broad MeSH descriptors, but the analysis we performed by deactivating the explosion for the gold standard (using *[mesh:noexp]*) revealed similar ranking of the four strategies and similar variability (data not shown). Moreover, a new version of PubMed is available since November 2019 and provides a modified version of the ATM. Unfortunately, the details about its modifications have not been published at the time of this study, and therefore, it is not reproducible [27]. Future research could analyze the new version of the ATM in comparison with the expansion strategies and with the legacy ATM.

Our results suggest that the choice of the semantic expansion strategy used to build the query must be made according to the descriptor. Since the automatic assessment tested here allowed assessment of all the MeSH preferred descriptors, it is now possible to choose which semantic expansion strategy to use to build a query for a given descriptor, according to the performances of the three metrics precision, recall, and F-measure. As the processing time of this automatic assessment is quite low, it can be updated frequently (each day, each week, or each month). Technically, the assessment could also be performed in real time, although this does not seem necessary since the results should not vary greatly during short periods of time.

The availability of quantitative measurements of the performances of different strategies now allows users to decide which semantic expansion to use given a particular MeSH descriptor. Depending on their specific needs, users could either choose the strategy providing the best precision, best recall, or best F-measure, since these performances could be accomplished by different strategies. These considerations led our team to develop an interface that allows users to input their MeSH preferred descriptor and to choose which metric they wish to maximize (precision, recall, or F-measure). This tool is freely available on the HeTOP website [28] (Multimedia Appendix 1). Users can search for a term, and the algorithm will try to match the term with the corresponding MeSH descriptor. Once on the description page of a descriptor, the “PubMed/Doc’CISMef” tab needs to be selected to go to the query building section. There, in section 2 “options,” users can select which metric they want to maximize (precision, recall, or F-measure). The algorithm will determine which expansion strategy yields the best score for the metric and automatically construct the query with the corresponding semantic expansion. Thereafter, in section 3 “queries,” a button with a PubMed icon appears with a tag corresponding to the semantic expansion that will be used to build the query. By clicking this button, the query will be automatically built and sent to the PubMed search engine in a new browser page. A perspective could be to go even further in the customization of the queries, with the possibility to add each synonym successively, each time assessing in real time the performance of this custom query.

### Limitations

This study has some limitations. First, in order to assess the metrics in an automatic manner, the scope of the search had to be restricted to the indexed citations of the MEDLINE database. The assessment of recent nonindexed citations could only be performed manually, with all the limiting factors previously described in the literature. However, it is legitimate to assume that the different semantic expansions would perform in the same way for the entire database since there is no reason to think that the indexing paradigms would shift suddenly for a given descriptor. Moreover, the results presented here are consistent with manual evaluations of previous studies, suggesting there is no major bias in this new methodology. Second, the queries built were simple queries based on only one MeSH preferred term. It would be necessary to evaluate the performances of these different semantic expansions with more complex queries, associating multiple MeSH preferred terms. However, the behavior of such queries would be identical because the semantic expansion of each term would be treated independently and then recombined with Boolean operators, which is the default behavior of PubMed’s ATM.

### Conclusions

In this study, we present an innovative method to automatically compute for PubMed citations the three main metrics used in information science. This new method allowed us to compare four semantic expansion strategies to query PubMed on all MeSH descriptors. The results confirmed great variability depending on the descriptor. Hence, there is a need to propose to users the semantic expansion that best fits their specific objectives. Owing to the possibility of regularly updating the performances of these search strategies for all MeSH descriptors, our team has developed an interface that allows users to input a descriptor and then proposes the best semantic expansion to maximize either precision, recall, or F-measure.

## Appendix

Multimedia Appendix 1 Screenshots explaining how to use the automatic query building tool on the HeTOP website.

## Abbreviations

ATM: automatic term mapping
CISMeF: Catalogue et Index des Sites Médicaux de langue Française
CUI: concept unique identifier
HeTOP: Health Terminology/Ontology Portal
KOS: knowledge organization system
MeSH: Medical Subject Headings
NLM: National Library of Medicine
UMLS: Unified Medical Language System
