# Supplementary material for: Identification of the Best Semantic Expansion to Query PubMed Through Automatic Performance Assessment of Four Search Strategies on All Medical Subject Heading Descriptors: Comparative Study
Source: JMIR Med Inform. 2020 Jun 4;8(6):e12799. doi: 10.2196/12799 (PMC7303830; doi:10.2196/12799)
Supplement: Multimedia Appendix 1 [file medinform_v8i6e12799_app1.pdf]

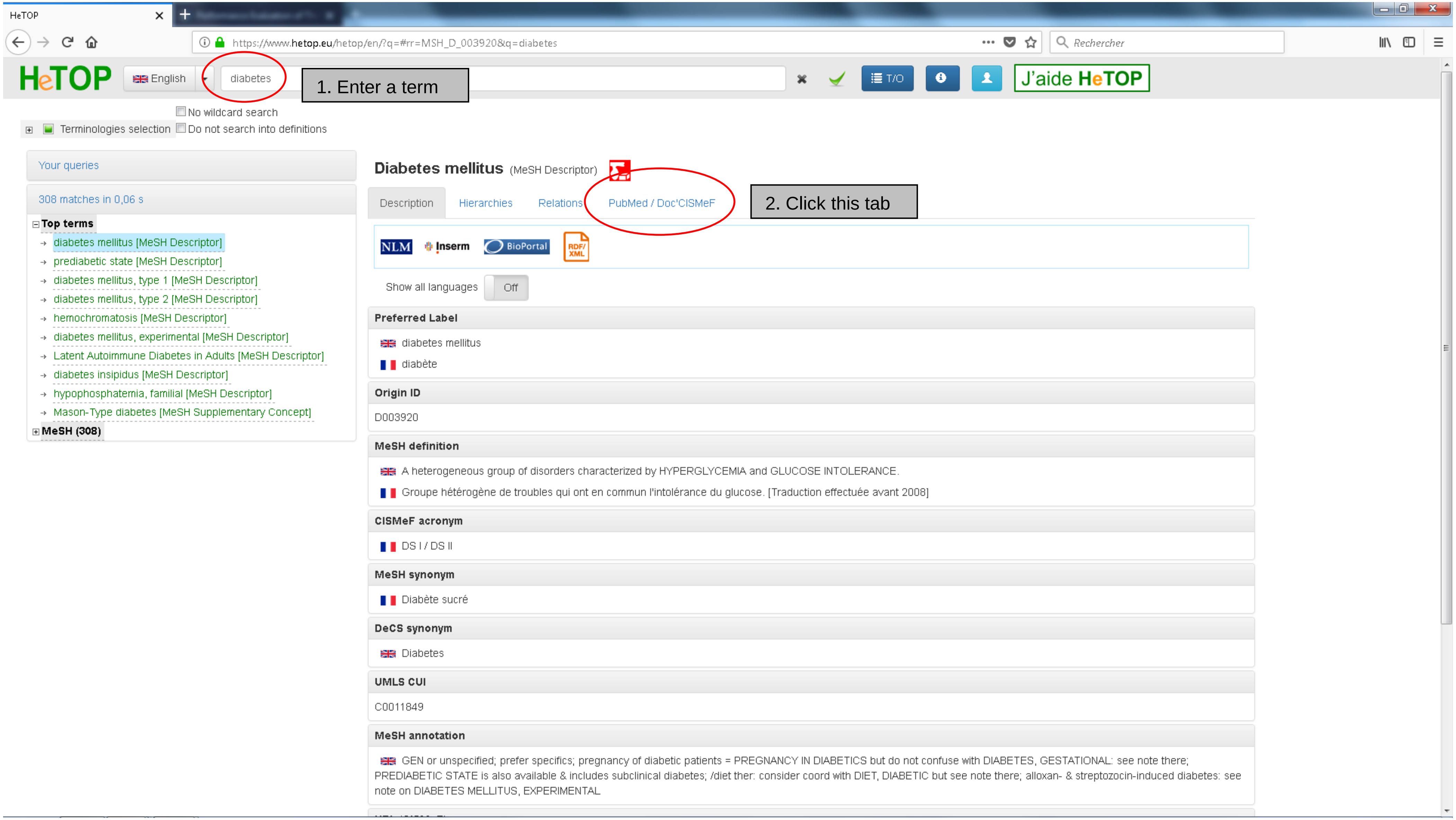
HeTOP
https://www.hetop.eu/hetop/en/?q=#rr=MSH_D_003920&q=diabetes
Rechercher
HeTOP
English
diabetes
1. Enter a term
T/O
J'aide HeTOP
No wildcard search
Terminologies selection
Do not search into definitions
Your queries
308 matches in 0,06 s
Top terms
diabetes mellitus [MeSH Descriptor]
prediabetic state [MeSH Descriptor]
diabetes mellitus, type 1 [MeSH Descriptor]
diabetes mellitus, type 2 [MeSH Descriptor]
hemochromatosis [MeSH Descriptor]
diabetes mellitus, experimental [MeSH Descriptor]
Latent Autoimmune Diabetes in Adults [MeSH Descriptor]
diabetes insipidus [MeSH Descriptor]
hypophosphatemia, familial [MeSH Descriptor]
Mason-Type diabetes [MeSH Supplementary Concept]
MeSH (308)
Diabetes mellitus (MeSH Descriptor)
Description
Hierarchies
Relations
PubMed / Doc'CISMeF
2. Click this tab
NLM
Inserm
BioPortal
RDF/XML
Show all languages
Off
Preferred Label
diabetes mellitus
diabète
Origin ID
D003920
MeSH definition
A heterogeneous group of disorders characterized by HYPERGLYCEMIA and GLUCOSE INTOLERANCE.
Groupe hétérogène de troubles qui ont en commun l'intolérance du glucose. [Traduction effectuée avant 2008]
CISMeF acronym
DS I / DS II
MeSH synonym
Diabète sucré
DeCS synonym
Diabetes
UMLS CUI
C0011849
MeSH annotation
GEN or unspecified; prefer specifics; pregnancy of diabetic patients = PREGNANCY IN DIABETICS but do not confuse with DIABETES, GESTATIONAL: see note there; PREDIABETIC STATE is also available & includes subclinical diabetes; /diet ther: consider coord with DIET, DIABETIC but see note there; alloxan- & streptozocin-induced diabetes: see note on DIABETES MELLITUS, EXPERIMENTAL

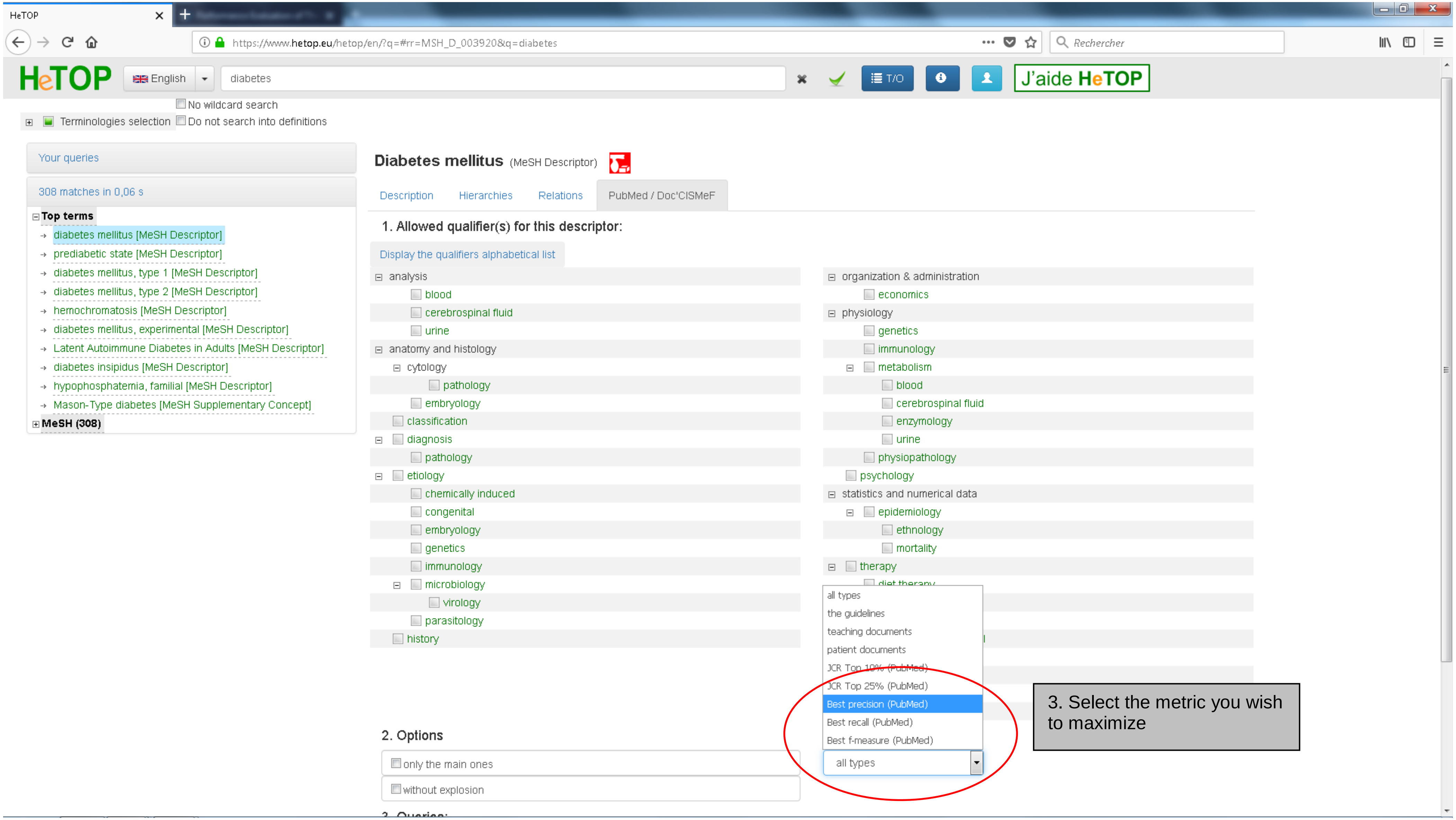
HeTOP
https://www.hetop.eu/hetop/en/?q=#rr=MSH_D_003920&q=diabetes
Rechercher
HeTOP
English
diabetes
T/O
J'aide HeTOP
No wildcard search
Terminologies selection
Do not search into definitions
Your queries
308 matches in 0,06 s
Top terms
diabetes mellitus [MeSH Descriptor]
prediabetic state [MeSH Descriptor]
diabetes mellitus, type 1 [MeSH Descriptor]
diabetes mellitus, type 2 [MeSH Descriptor]
hemochromatosis [MeSH Descriptor]
diabetes mellitus, experimental [MeSH Descriptor]
Latent Autoimmune Diabetes in Adults [MeSH Descriptor]
diabetes insipidus [MeSH Descriptor]
hypophosphatemia, familial [MeSH Descriptor]
Mason-Type diabetes [MeSH Supplementary Concept]
MeSH (308)
Diabetes mellitus (MeSH Descriptor)
Description
Hierarchies
Relations
PubMed / Doc'CISMeF
1. Allowed qualifier(s) for this descriptor:
Display the qualifiers alphabetical list
analysis
blood
cerebrospinal fluid
urine
anatomy and histology
cytology
pathology
embryology
classification
diagnosis
pathology
etiology
chemically induced
congenital
embryology
genetics
immunology
microbiology
virology
parasitology
history
organization & administration
economics
physiology
genetics
immunology
metabolism
blood
cerebrospinal fluid
enzymology
urine
physiopathology
psychology
statistics and numerical data
epidemiology
ethnology
mortality
therapy
all types
the guidelines
teaching documents
patient documents
JCR Top 25% (PubMed)
Best precision (PubMed)
Best recall (PubMed)
Best f-measure (PubMed)
all types
2. Options
only the main ones
without explosion
3. Select the metric you wish to maximize

HeTOP | English | diabetes | T/O | J'aide HeTOP

- diabetes mellitus, type 1 [MeSH Descriptor]
- diabetes mellitus, type 2 [MeSH Descriptor]
- hemochromatosis [MeSH Descriptor]
- diabetes mellitus, experimental [MeSH Descriptor]
- Latent Autoimmune Diabetes in Adults [MeSH Descriptor]
- diabetes insipidus [MeSH Descriptor]
- hypophosphatemia, familial [MeSH Descriptor]
- Mason-Type diabetes [MeSH Supplementary Concept]

MeSH (308)

- analysis
  - blood
  - cerebrospinal fluid
  - urine
- anatomy and histology
  - cytology
    - pathology
  - embryology
- classification
- diagnosis
  - pathology
- etiology
  - chemically induced
  - congenital
  - embryology
  - genetics
  - immunology
  - microbiology
    - virology
  - parasitology
- history
- organization & administration
  - economics
- physiology
  - genetics
  - immunology
  - metabolism
    - blood
    - cerebrospinal fluid
    - enzymology
    - urine
  - physiopathology
- psychology
- statistics and numerical data
  - epidemiology
    - ethnology
    - mortality
- therapy
  - diet therapy
  - drug therapy
  - nursing
  - prevention and control
  - radiotherapy
  - rehabilitation
  - surgery
- veterinary

2. Options

- only the main ones
- without explosion

Best precision (PubMed)

3. Queries:

PubMed ATM

4. Click the icon to automatically build the query and send it to PubMed

You may also use the Query Builder:

CRBM
Constructeur de Requêtes Bibliographiques Médicales

© Copyright 2010-2020 Rouen University Hospital
Terms - Contact
